# Nano vitamin E improved the antioxidant capacity of broiler chickens

**DOI:** 10.1093/jas/skae095

**Published:** 2024-04-29

**Authors:** Jinghui Zhou, Zhipeng Li, Wei Guo, Yibing Wang, Rui Liu, Xinhuo Huang, Yingge Li, Xiaojun Yang, Le Liu, Yanli Liu, Xiurong Xu

**Affiliations:** College of Animal Science and Technology, Northwest A&F University, Yangling 712100, China; College of Animal Science and Technology, Northwest A&F University, Yangling 712100, China; College of Animal Science and Technology, Northwest A&F University, Yangling 712100, China; College of Animal Science and Technology, Northwest A&F University, Yangling 712100, China; College of Animal Science and Technology, Northwest A&F University, Yangling 712100, China; Nano Vitamin Engineering Research Center, Xi’an 710000, China; Shaanxi Province Animal Husbandry Technology Extension Station, Xi’an710000, China; College of Animal Science and Technology, Northwest A&F University, Yangling 712100, China; Department of Biology, University of Massachusetts Amherst, Amherst, MA, USA; College of Animal Science and Technology, Northwest A&F University, Yangling 712100, China; College of Animal Science and Technology, Northwest A&F University, Yangling 712100, China

**Keywords:** antioxidant capacity, broiler, nano vitamin E, pharmacokinetics, stress

## Abstract

Vitamin E (VE) is a potent nutritional antioxidant that is critical in alleviating poultry oxidative stress. However, the hydrophobic nature and limited stability of VE restrict its effective utilization. Nanotechnology offers a promising approach to enhance the bioavailability of lipophilic vitamins. The objective of this experiment was to investigate the effects of different sources and addition levels of VE on the growth performance, antioxidant capacity, VE absorption site, and pharmacokinetics of Arbor Acres (AA) broilers. Three hundred and eighty-four 1-d-old AA chicks were randomly allocated into four groups supplemented with 30 and 75 IU/kg VE as regular or nano. The results showed that dietary VE sources had no significant impact on broiler growth performance. However, chickens fed 30 IU/kg VE had a higher average daily gain at 22 to 42 d and 1 to 42 d, and lower feed conversion ratio at 22 to 42 d than 75 IU/kg VE (*P* < 0.05). Under normal feeding conditions, broilers fed nano VE (NVE) displayed significantly higher superoxide dismutase (SOD) activity and glutathione peroxidase (GSH-Px) enzyme activities and lower malonic dialdehyde (MDA) concentration (*P* < 0.05). Similarly, NVE had a higher antioxidant effect in the dexamethasone-constructed oxidative stress model. It was found that nanosizing technology had no significant effect on the absorption of VE in the intestinal tract by examining the concentration of VE in the intestinal tract (*P* > 0.05). However, compared to broilers perfused with regular VE (RVE), the NVE group displayed notably higher absorption rates at 11.5 and 14.5 h (*P* < 0.05). Additionally, broilers perfused with NVE showed a significant increase in the area under the concentration versus time curve from zero to infinity (AUC_0−∞_), mean residence time (MRT_0−∞_), elimination half-life (t_1/2z_), and peak concentration (C_max_) of VE in plasma (*P* < 0.05). In summary, nanotechnology provides more effective absorption and persistence of VE in the blood circulation for broilers, which is conducive to the function of VE and further improves the antioxidant performance of broilers.

## Teaser

Nanotechnology significantly enhanced the absorption efficiency of VE ensuring its prolonged circulation and thereby boosts the antioxidant capabilities in broilers.

## Introduction

Poultry plays a crucial role in global meat production, and to meet the growing demand for animal protein, it is imperative to maximize poultry yield (Tona, 2018). With the advancement of high-density and intensive feeding practices, stress, especially oxidative stress, has become one of the limitations to efficient production in the broiler industry (Elitok, 2018). Oxidative stress places poultry in a suboptimal health state, where excessive oxygen free radicals can disrupt the mucosal barriers of organs, resulting in impaired physiological functions, increased susceptibility to diseases, and a decline in production and reproductive performance, as well as the quality of animal products, significantly impacting economic efficiency in production (Zou et al., 2017). To mitigate the consequences of oxidative stress, antioxidants and micronutrients are commonly incorporated into animal feed (Akinyemi and Adewole, 2021). Dietary supplementation of selenium, vitamin E (VE; α-tocopherol), and carotenoids can regulate the antioxidant defense of poultry (Surai et al., 2016). Among them, VE is the main chain-breaking antioxidant in cells and is considered to be the “headquarters” of the antioxidant defense network, which reduces the risk of free radical-induced lipid peroxidation damage to cells and tissues (Voljč et al., 2011). Cheng et al. (2017) reported that supplementation of VE in the diet increased the levels of superoxide dismutase (SOD), total antioxidant (T-AOC), glutathione peroxidase (GSH-Px) in serum and liver and decreased the levels of malondialdehyde (MDA), thus improving the antioxidant capacity of cyclophosphamide-treated broilers. Liu et al. (2019) showed that VE supplementation promoted a balance between antioxidants and oxidants, which ameliorated oxidative stress in attacked Salmonella laying hens. Gao et al. (2010) showed that VE supplementation alleviated oxidative stress induced by dexamethasone (DEX) treatment and improved the growth performance of broilers. Conversely, VE deficiency deteriorated the redox state of chicken muscle (Avanzo et al., 2001). However, as a lipid-soluble vitamin, the hydrophobicity of VE may present significant challenges for adequate VE transport and uptake (Brigelius-Flohé et al., 2002), such as poor water solubility (Sagalowicz and Leser, 2010) and low bioavailability (Yang and McClements, 2013), which limits the effectiveness of VE as an oral supplement, and higher supplemental doses are required to maintain effective levels. Furthermore, both natural and synthetic VE easily cause degradation during various stages of feed processing, manufacturing, and storage, posing challenges for their utilization as feed additives. To address these issues, more stable formulations have been developed. To enhance stability, the phenolic moiety of alpha-tocopherol is converted into an ester using acetic or succinic acid, producing commercial products such as alpha-tocopherol acetate or alpha-tocopherol succinate. Among these formulations, all-rac α-tocopheryl acetate is widely used in animal feed supplementation due to its superior stability and cost-effectiveness. However, it still faces encounters limitations regarding bioavailability in the bodies of poultry (Desmarchelier et al., 2013).

Therefore, a lot of efforts have been made to enhance the utilization and integration of VE into food products, aiming to develop effective delivery systems that safeguard VE from chemical degradation and enhance its bioavailability upon ingestion (Yang and McClements, 2013). Nano-emulsions have gained attention in food applications, due to good oral bioavailability and the potential for robust and transparent drug delivery systems (Golfomitsou et al., 2018). Salvia-Trujillo et al. (2017) found that smaller-sized lipid droplets were digested more rapidly in simulated gastrointestinal fluids, suggesting that mixed micelles of solubilized lipophilic vitamins may be formed more rapidly in the small intestine. In recent years, the rise of nanotechnology has generated significant interest in nano vitamin E (NVE) due to its unique properties such as large surface area, high surface activity, high dissolution rate, high absorption rate, and high utilization rate (Sekhon, 2010). Alqahtani et al. (2015) demonstrated that encapsulating α-tocopherol (α-T) and a tocotrienol-rich fraction in PLGA or PLGA/chitosan nanoparticles enhanced the uptake of α-T and tocotrienol-rich fraction by Caco2 cells without inducing toxicity, in contrast to the control group. In addition, NVE has been reported to have a higher absorption rate in rats compared with regular VE (RVE; Saratale et al., 2018). Therefore, the objective of this experiment was to evaluate the effects of different dietary VE sources and levels on growth performance, antioxidant capacity, site of VE absorption as well as pharmacokinetics of Arbor Acres (AA) broilers.

## Materials and Methods

In this study, all applications were subject to the Institutional Animal Conservation and Utilization Committee of the Northwest A&F University, under Permit Number NWAFAC 1,008. Chickens were obtained from Xi’an Dacheng Poultry Co., Ltd. (Xi’an, Shaanxi, China) and they were housed in an environmentally controlled facility.

### Experimental birds, diets, and design

Three hundred and eighty-four 1-d-old AA broilers (192 male and 192 female) were randomly divided into four treatment groups with six replicates, each replicate contained 16 chickens (eight male and eight female). The four treatments consisted of a 2 × 2 factorial arrangement of two VE sources (RVE or NVE) and two levels (30 or 75 IU/kg) of each VE source. RVE was obtained from Golden Crown, a commercial feed ingredient supplier (Shaanxi Golden Crown Biotechnology Co., Ltd.). NVE was obtained by mixing an emulsifier with commercial RVE in proportion to reduce the RVE size (Shaanxi Golden Crown Biotechnology Co., Ltd.). Dietary VE supplementation levels were set to create treatment diets containing recommended or moderate excessive levels of VE, based on the feeding standards of the surrounding broiler plant. To reduce the VE contribution of the basal diet in treatment diets, VE was removed from the vitamin premix. Based on the nutritional requirements recommended by the Chinese Standard for Chicken Feed (NY/T 33-2004), a corn–soybean meal type basal diet was formulated (Table 1). Throughout the experiment, chickens were fed and watered ad libitum. Lighting, relative humidity, and temperature were maintained according to the guidelines for AA broilers.

**Table 1.** Composition and nutrient content of the basal diet

Items	0 to 21 d	22 to 42 d
*Ingredients, %*		
Corn	53.78	58.89
Soybean meal	25.76	17.20
Corn DDGS	5.00	5.00
Corn protein flour	4.00	5.00
Cottonseed meal	3.95	4.00
Soybean oil	3.01	4.80
Limestone	1.60	1.40
CaHPO4	1.42	1.16
l-Lys	0.50	0.54
dl-Met	0.27	0.20
NaCl	0.26	0.26
Multi-minerals^1^	0.15	0.15
l-Thr	0.12	0.11
l-Try	0.08	0.19
Choline chloride	0.08	0.08
Multi-vitamin^2^	0.02	0.02
Total	100.00	100.00
*Calculated nutrient levels*		
Metabolic energy, kcal/kg	2,900.00	3,090.00
Crude protein, %	21.65	19.10
Crude fat, %	5.20	7.24
Crude fiber, %	3.18	2.77
Calcium, %	0.95	0.81
Total phosphorus, %	0.62	0.56
Available phosphorus, %	0.41	0.36
Total Lys, %	1.34	1.19

^1^The premix provided the following per kg of diets: Cu 8.4 mg, Fe 54 mg, Zn 49.5 mg, and Mn 70.5 mg.

^2^The premix provided the following per kg of diets: (1 to 42 d) VA 11,200 IU, VD_3_ 3,360 IU, VK_3_ 4.0 mg, VB_1_ 2.2 mg, VB_2_ 7.28 mg, and VB_6_ 4.8 mg.

At 21 d of age, all broilers were weighed after fasting for 12 h. Six male healthy broilers with average body weight (BW) were selected from each dietary treatment. Blood samples were obtained from the veins of the wings and transferred to vacuum tubes. The tubes were centrifuged at 1,500 × g for 15 min to obtain plasma samples; then, the plasma was collected immediately and stored at −20 °C for further analysis. After blood collection, birds were euthanized by cervical dislocation. Organization samples were collected from the liver, spleen, bursa, and thymus and weighed immediately.

To compare the effects of RVE and NVE on the antioxidant status of broiler chickens, oxidative stress was induced by DEX. At 29 d of age, six male AA broilers with similar body weights were selected from the remaining breeds in each group. DEX solution was injected subcutaneously into the abdomen at a dose of 3 mg/kg BW on days 29, 31, and 33. At 33 d of age, blood samples were collected 4 and 8 h after subcutaneous DEX injection. After blood collection, birds were euthanized by cervical dislocation.

At 42 d of age, six male AA chickens with a nearly average BW were randomly selected from the remaining chickens in each diet treatment group. As mentioned earlier, blood samples were collected from each chicken, and the plasma obtained after centrifugation of the blood samples was stored at −20 °C for further analysis. After blood collection, birds were euthanized by cervical dislocation. Immediately after killing the chickens, the liver, spleen, bursa of Fabricius, and thymus were weighed. Chyme in the duodenum, jejunal, and ileal lumen were collected and stored at −80 °C for further analysis.

In addition, to investigate whether the nano-treatment improves the pharmacokinetic properties of VE in broilers and thus the antioxidant properties. A total of 16 35-d-old male AA broilers were randomly divided into two treatment groups; each group received a 1 mL infusion of either 20 IU/mL RVE or NVE. Blood was collected at 1 h before VE perfusion and at 1, 4, 5.5, 8.5, 11.5, and 14.5 h after infusion.

### Determination of VE in plasma and intestinal chyme

The plasma and intestinal chyme VE concentration was determined using the corresponding commercial test kits according to the manufacturer’s instructions (A008-1-1, VE assay kit, Nanjing Jiancheng Bioengineering Institute, Nanjing, China).

### Determination of antioxidant enzymes in serum

The activity of serum antioxidant enzymes was determined by measuring T-AOC, SOD, GSH- Px, and MDA levels. Analyzed using commercially available analytical kits from Nanjing Jiancheng Bioengineering Institute, Nanjing, China (used according to the manufacturer’s instructions).

### Pharmacokinetic analyses

Approximately 5 mL of blood samples were collected from the wing vein into 10 mL heparin plastic centrifuge tubes at 1 h before VE perfusion and at 1, 4, 5.5, 8.5, 11.5, and 14.5 h after infusion. Plasma was separated immediately by centrifugation at 1,500 × *g* for 15 min and stored at below −20 °C until analysis. The plasma VE concentration was determined using the corresponding commercial test kits according to the manufacturer’s instructions (A008-1-1, VE assay kit, Nanjing Jiancheng Bioengineering Institute, Nanjing, China).

Concentration-time curves were generated using GraphPad Prism 8 software (GraphPad Software, San Diego, CA, USA) based on the plasma concentration data. The pharmacokinetic parameters calculated included the maximum concentration of VE (C_max_), the time taken to reach peak concentration (T_max_), the area under the concentration-time curve from zero to the last measured time point (AUC_0−t_), the area under the concentration-time curve from zero to infinity (AUC_0−∞_), and the elimination half-life (t_1/2z_).

### Statistical Analysis

The data were analyzed using SPSS version 26.0 (IBM Corp., Chicago, IL, USA). Chicken performance and antioxidants were analyzed as a 2 × 2 factorial arrangement of treatments by two-way analysis of variance, with a model including the main effects of VE source, VE level, and their interaction. Differences with a *P* value of < 0.05 were considered significant.

The effects of the two VE sources (RVE vs. NVE) on VE concentrations in bird plasma were compared using an independent sample *t*-test (Student’s *t*-test), and differences with a *P* value of < 0.05 were considered significant.

## Results

### Growth performance and relative organ weights

During the 1 to 21, 22 to 42, and 1 to 42 d periods, dietary VE sources had no significant effects on ADFI, average daily gain (ADG), and feed conversion ratio (FCR; Table 2). However, when comparing the 75 IU/kg VE group to the 30 IU/kg VE group, the dietary supplementation of 30 IU/kg VE significantly increased ADG at 22 to 42 d and 1 to 42 d and decreased FCR at 22 to 42 d (*P* < 0.05).

**Table 2.** Effects of vitamin E sources and inclusion levels in diets on growth performance of chicken broilers

Period	Items	RVE		NVE		SEM^1^	*P*-value		
		30 IU/kg	75 IU/kg	30 IU/kg	75 IU/kg		Source	Level	Source × level
1 to 21 d	ADFI(g)	48.069	48.152	48.775	47.789	0.293	0.783	0.470	0.393
	ADG(g)	33.196	33.163	33.668	33.048	0.258	0.748	0.557	0.597
	FCR(g)	1.449	1.452	1.449	1.447	0.004	0.761	0.964	0.731
22 to 42 d	ADFI(g)	120.106	118.041	117.257	113.812	1.250	0.179	0.290	0.787
	ADG(g)	67.585	65.996	67.918	61.995	0.952	0.310	0.048	0.233
	FCR(g)	1.781	1.790	1.727	1.859	0.017	0.795	0.025	0.047
1 to 42 d	ADFI(g)	82.920	81.750	81.754	79.127	0.662	0.161	0.160	0.579
	ADG(g)	51.184	50.229	51.419	47.988	0.499	0.268	0.024	0.176
	FCR(g)	1.621	1.628	1.590	1.650	0.009	0.802	0.072	0.148

^1^ *n* = 6.

ADFI, average daily feed intake; ADG, average daily gain; FCR, feed conversion ratio; RVE, regular vitamin E; NVE, nano vitamin E; SEM, standard error of mean.

On 21 d, the immune organ index was not influenced by dietary VE inclusion levels or sources (*P* > 0.05). However, at 42 d, the main effects of VE sources, inclusion levels, and their interaction were significant for relative organ weights (Table 3). Chickens fed RVE had higher relative liver weights compared to those fed diets containing NVE (*P *< 0.05), and the liver relative weight in the 75 IU/kg VE supplementation group was greater than that in the 30 IU/kg group (*P* < 0.05). The relative weight of the thymus was not affected by VE sources. However, as the dietary inclusion levels of VE increased, there was a decrease in the relative weight of the thymus (*P* < 0.05). In addition, the organ weight of bursae of Fabricius was affected by interaction between dietary VE level and source (*P* < 0.05).

**Table 3.** Effects of vitamin E sources and inclusion levels in diets on the immune organs of chicken broilers

Period	Items	RVE		NVE		SEM^1^	*P*-value		
		30 IU/kg	75 IU/kg	30 IU/kg	75 IU/kg		Source	Level	Source × level
1 to 21 d	Liver	27.401	26.302	27.499	24.797	0.638	0.588	0.153	0.538
	Thymus	1.727	1.494	1.871	1.630	0.076	0.367	0.132	0.978
	Spleen	0.989	1.057	1.087	1.002	0.042	0.811	0.928	0.392
	Bursa of fabricius	3.132	3.230	2.626	2.613	0.166	0.106	0.899	0.868
1 to 42 d	Liver	18.939	23.358	18.408	18.692	0.617	0.010	0.018	0.034
	Thymus	1.946	1.109	2.341	1.152	0.202	0.554	0.011	0.634
	Spleen	1.002	1.431	1.045	1.278	0.112	0.811	0.162	0.673
	Bursa of fabricius	1.828	1.117	1.519	1.650	0.103	0.561	0.142	0.038

^1^ *n* = 6.

SEM, standard error of mean

### Serum antioxidant enzyme activities before DEX injection

**Figure 1.**
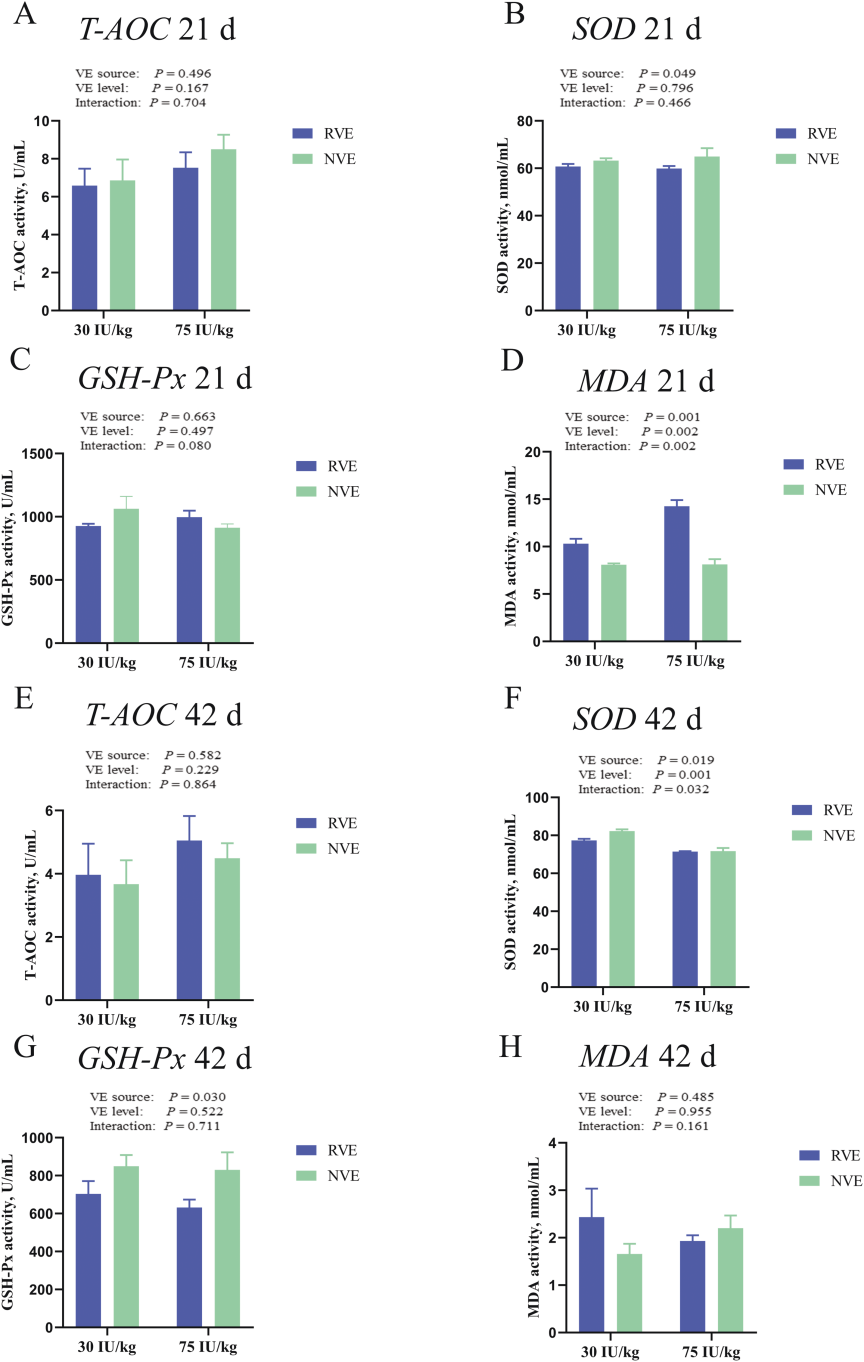
Effects of different VE sources and inclusion levels on antioxidant enzyme activities of broilers fed experimental diets at 21 d (A–D) and 42 d of age (E-H). (A) T-AOC, (B) SOD, (C) GSH-Px, (D) MDA, (E) T-AOC, (F) SOD, (G) GSH-Px, (H) MDA. *P* values mean the significance from 2 × 2 factorial analysis (sources and inclusion levels).

Figure 1 illustrates the impact of dietary supplementation with different VE sources and inclusion levels on antioxidant enzyme activity in chickens. On 21 d, SOD activity and MDA concentration were found to be influenced by dietary VE sources (*P* < 0.05). Chickens fed diets containing NVE had higher SOD activity (*P *< 0.05) and lower MDA content (*P* < 0.05) in their serum compared to those fed diets containing RVE. Furthermore, the MDA concentration in serum was also influenced by inclusion levels of VE in diets, with the concentration of MDA in the 30 IU/kg VE group being lower than that in the 75 IU/kg VE group (*P* < 0.05). In contrast, T-AOC and GSH-Px activity remained unaffected by dietary VE levels or sources (*P* > 0.05). However, on 42 d, different VE sources and inclusion levels had influenced SOD activity but not MDA concentration in serum. Birds fed NVE had greater SOD activity than birds fed diets containing RVE (*P* < 0.05). SOD activity was reduced with the increase of VE concentration in diet (*P* < 0.05). Besides, chickens fed NVE diets had greater GSH-Px activity than chickens fed diets containing RVE; however, different VE inclusion levels had no effect on GSH-Px activity in serum (*P* < 0.05).

### Serum antioxidant enzyme activities after DEX injection

Table 4 outlined the effects of different sources and levels of VE supplementation on antioxidant enzyme activities in broilers exposed to oxidative stress induced by DEX. Under oxidative stress conditions, at 4 and 8 h after subcutaneous injection of DEX at the age of 33 d, the activity of T-AOC and the concentration of MDA in serum were influenced by the source of VE (*P* < 0.05). Chicken-fed NVE had a lower concentration of MDA compared with those fed diets containing RVE (*P* < 0.05), and the concentration of MDA was lower in the 30 IU/kg VE group compared with the 75 IU/kg VE group.

**Table 4.** Effects of dietary supplementation with different VE sources and inclusion levels to resistance dexamethasone-induced oxidative stress in chicken

Period	Items	RVE		NVE		SEM^1^	*P*-value		
		30 IU/kg	75 IU/kg	30 IU/kg	75 IU/kg		Source	Level	Source × level
4h	T-AOC	9.820	12.847	5.695	10.748	0.712	0.005	0.001	0.319
	MDA	28.580	11.195	11.580	9.674	2.435	0.023	0.018	0.052
	GSH-Px	1,949.010	1,795.813	1,982.970	1,904.120	56.409	0.562	0.350	0.761
8h	T-AOC	9.168	8.494	12.113	9.733	0.513	0.048	0.136	0.391
	MDA	15.830	10.305	10.028	7.415	0.972	0.014	0.020	0.374
	GSH-Px	1,408.458	1,519.808	1,424.458	1,500.820	33.469	0.983	0.202	0.806

^1^ *n* = 6.

T-AOC, total antioxidant capacity; MDA, malonic dialdehyde; SOD, superoxide dismutase; GSH-Px, glutathione peroxidase; SEM, standard error of mean.

### Intestinal VE uptake in chicken

**Figure 2.**
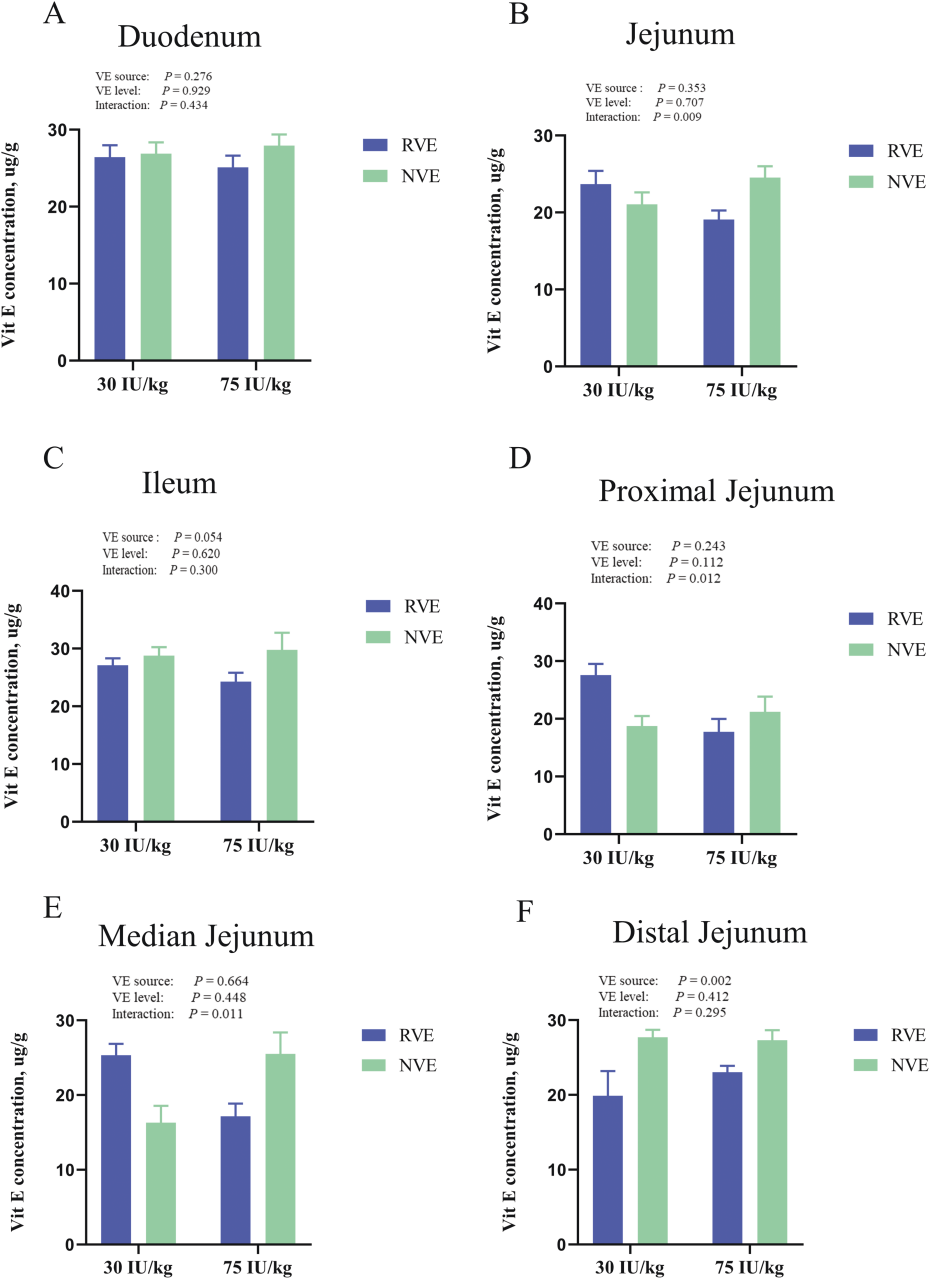
Vitamin E content in intestinal fragments of chickens at 42 d of age after feeding with different VE sources and levels. (A) Concentration of VE in duodenal chyme, (B) Concentration of VE in jejunal chyme, (C) Concentration of VE in ileal chyme. (D) Proximal jejunum VE concentration, (E) Median jejunum VE concentration, and (F) Distal jejunum VE concentration. *P* values mean the significance from 2 × 2 factorial analysis (sources and inclusion levels).

To investigate whether nano VE enhanced antioxidant properties by improving the absorption and utilization of VE, the concentrations of VE were detected in different sites of intestinal chyme of broilers. The change in VE concentration were used to determine the main absorption site of VE in the broiler intestine (Figure 2). The results showed that VE source, inclusion levels, and their interaction had no significant effect on the absorption of VE in the intestine (*P* > 0.05). Furthermore, we examined the content of VE in the proximal, median, and distal chyme of jejunum. In the digest of the proximal, and median jejunum, VE source, inclusion levels, and their interaction had no significant effect on VE absorption, whereas different VE sources affected VE absorption in the distal jejunum. The VE content in chyme of NVE supplementation groups was significantly higher than in the RVE group (*P* < 0.05).

### Nanotechnology affects the pharmacokinetics of VE in broilers

**Figure 3.**
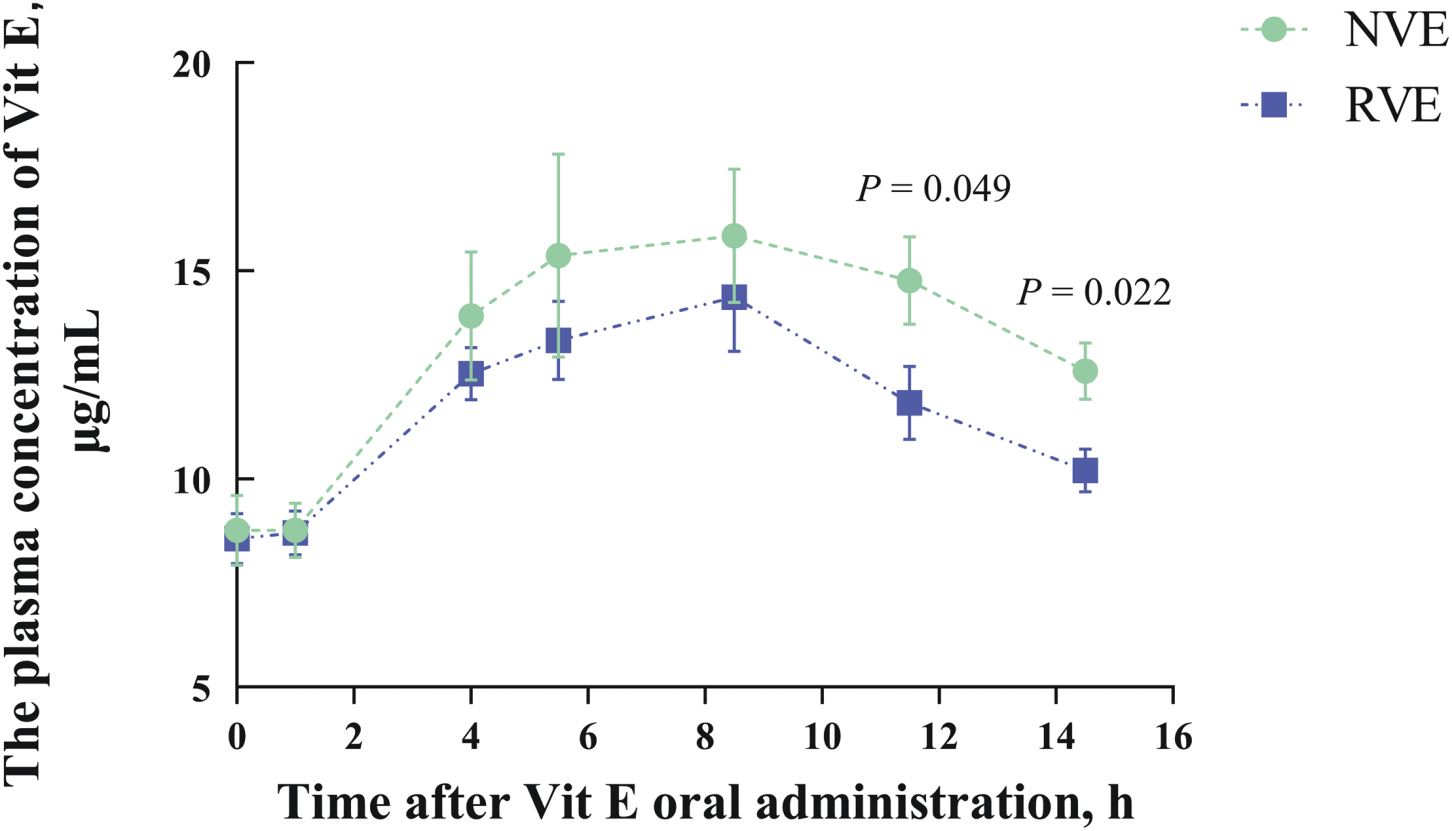
Mean ± SD plasma concentrations (μg/mL) of vitamin E in the RVE and NVE after a single administration of vitamin E (20 IU) in broilers. RVE, 20 IU regular VE; NVE, 20 IU nano VE.

The results of the blood concentration-time curve (Figure 3) showed that the NVE group maintained higher blood concentrations at 4, 5.5, and 8.5 h after infusion (*P* > 0.05), and the absorption rate at 11.5 and 14.5 h was significantly higher than that in the RVE group (*P* < 0.05). Relevant pharmacokinetic parameters were calculated using DAS 3.0 (Table 5). NVE treatment significantly increased the AUC_0−∞_, MRT_0−∞_, t_1/2z_, and C_max_ of VE in broiler plasma compared to the RVE group (*P* < 0.05). However, there were no significant differences in AUC_0−t_, MRT_0−t_, or T_max_ (*P* > 0.05). Furthermore, compared with RVE, the bioavailability of NVE in broilers increased by 146.52% according to software system fitting.

**Table 5.** Comparison of mean pharmacokinetic parameters between the RVE and NVE after a single administration of vitamin E (20 IU) in broilers

Parameters^1^	RVE^2^	NVE^3^
AUC_0−t_, μg•h/mL	174.797 ± 19.244	197.662 ± 36.911
AUC_0−∞_, μg•h/mL	343.253 ± 125.667	580.442 ± 236.579
MRT_0−t_, h	7.453 ± 0.317	7.628 ± 0.222^*^
MRT_0−∞_, h	18.163 ± 3.199	23.728 ± 3.233^*^
t_1/2z_, h	10.526 ± 3.204	14.843 ± 0.834^*^
T_max_, h	8.071 ± 2.070	8.500 ± 3.000
C_max_, μg/mL	16.096 ± 2.056	22.060 ± 4.581^*^
F, %	146.52	

^1^AUC_0−t_, area under the concentration versus time curve from zero to t; AUC_0−∞_, area under the concentration vs. time curve from zero to infinity; MRT, mean residence time; t_1/2z_, half-life of the elimination; T_max_, time to reach peak concentration; C_max_, peak concentration; F = [Ds × AUCtest/ Dtest × AUCs] × 100%, absolute bioavailability.

^2^RVE, perfusion 20 IU regular VE; NVE, perfusion 20 IU nano VE.

^3^Mean ± SD, *n* = 8, *P < 0.05, compared with RVE.

## Discussion

The increasing prevalence of stress has emerged as a significant constraint that negatively impacts poultry performance, product quality, and immune function, and is linked to increased oxidative stress caused by high temperature, intensive feeding, feed mildew, oxidation deterioration, excessive heavy metals, excessive or frequent use of antibiotics and other drugs, and diseases. VE supplementation helps reduce oxidative stress, but as a natural and potent antioxidant, the highly hydrophobic and low-stability properties of VE limit its application (Gawrysiak‐Witulska et al., 2009). Recently, there has been growing interest in utilizing nanotechnology to enhance the potency and bioavailability of VE (Mohd Zaffarin et al., 2020). Although several studies have demonstrated the potential benefits of nano VE in various fields, such as medicine and nutrition, its application in poultry remains relatively unexplored and lacks comprehensive research. This study was conducted to investigate the effects of VE source (regular vs. nano) and dietary inclusion level on growth performance, antioxidant capacity, and pharmacokinetics in AA broilers. We found that NVE improved the antioxidant properties of broilers by maintaining high levels of VE blood circulation for long periods of time without affecting the normal growth of the poultry.

The results showed that dietary VE sources had no significant effect on ADFI, ADG, and FCR. Consistent with the previous study, Pitargue et al. (2019) found that broiler growth performance was not affected by different VE sources in the diet. Similar results were found by Rey et al. (2015), whose study showed that the source of VE or the supplementation level had no impact on the growth performance of turkeys. However, in this study, broilers fed 30 IU/kg VE exhibited higher ADG at 22 to 42 d and 1 to 42 d and lower FCR at 22 to 42 d. It might be due to excess VE producing pro-oxidants rather than acting as antioxidants. Similarly, Englmaierová et al. (2011) reported the BW of 35-d-old broilers was significantly reduced when the birds were fed with a greater dietary VE level at 100 mg/kg under the non-stressed condition. Although the two concentrations were not sufficient to screen the recommended dose of NVE in broiler applications, our experimental results showed that the addition of 30 IU/kg and 75 IU/kg of NVE did not affect the normal development of broilers, providing a range of concentrations for the application of NVE in broiler diets. Further studies on the optimal concentration for using the nano form of VE as feed additives are needed.

The thymus, spleen, and bursa are important immune organs in broiler chickens, and their organ indexes can reflect the strength of immune function to a certain extent. The results of this study indicated that the weights of lymphoid organs were not affected by the sources of dietary VE. This is consistent with Niu et al. (2009), who reported that the source of VE in the broiler diet had no effect on the thymus, bursa, or spleen weight. However, the liver relative weight of the 75 IU/kg VE supplementation group was greater than the 30 IU/kg group. The reason for increasing liver weight by feeding high concentrations of VE may be due to the relatively high sensitivity of chickens to oxidative stress. Diets with high levels of antioxidants in the diet may increase oxidative stress and potentially inflammatory responses, which may lead to enlargement of the liver, as the liver is the most sensitive organ in the body to oxidative stress (Lu et al., 2014).

Changes in redox balance caused by endogenous and exogenous reactive oxygen species (ROS) are involved in a wide range of diseases, as well as being a phenomenon considered critical for survival. When ROS production exceeds antioxidant scavenging capacity, it reacts with polyunsaturated fatty acids in cell membranes, nucleotides in DNA, and key sulfhydryl bonds in proteins to cause tissue damage (Mittal et al., 2014). Antioxidant enzymes such as SOD and GSH-Px play an important role in preventing cell damage from ROS (Hu et al., 2015). SOD is effective in scavenging free radicals in vivo, and GSH-Px reduces the level of ROS by promoting the breakdown of H_2_O_2_ in vivo (Sabir et al., 2012). T-AOC is an important index of the antioxidant defense system, reflecting the ability of the non-enzymatic antioxidant defense system. Therefore, SOD, GSH-Px, and T-AOC are often used as effective indicators to objectively reflect the antioxidant status in animals (Zhao et al., 2016; Mohamed et al., 2019). In the present study, dietary supplementation with NVE increased serum SOD activity and GSH-Px content compared with RVE. The results showed that the supplementation of nano vitamins improved the antioxidant capacity of broiler chicks. A similar report was found by Ahmed et al. (2021), who reported that nano-emulsion VE enhanced the antioxidant activity of the liver and reduced lipid peroxidation. MDA is one of the main products of lipid peroxidation and its level reflects the degree of lipid peroxidation in the body (Selvakumar et al., 2005). In the present study, serum MDA levels were lower in chickens supplemented with NVE than in those supplemented with RVE. This reaction may be caused by the nanosizing of VE, which protects VE and ensures its nutritional value to animals. Due to microencapsulation, VE is dispersed and released more uniformly and consistently in the intestinal tract (Yoo et al., 2006). Thus, the microcapsule technology enhances the activity of antioxidant enzymes by improving digestion and absorption in the body (Valko et al., 2007).

To investigate whether the nano-treatment affects the efficiency of VE absorption in intestinal cells, this study examined VE content in intestinal fragments of chickens at 42 d of age after feeding with different VE sources and levels. Nano-treatment had no effect on the site of VE absorption but influenced the efficiency of VE absorption in the distal jejunum of chickens. This may be due to the fact that the nano-treatment protects the VE and ensures its nutritional value to the animal (Katouzian and Jafari, 2016). The nano-formulation likely facilitates enhanced permeation across the intestinal barrier, thus facilitating improved bioavailability and absorption of VE. Consequently, this may contribute to the enhancement of intestinal health, creating a favorable milieu for the optimal utilization of VE by the animals (Dawood et al., 2020).

To further investigate whether the improvement of the antioxidant performance of NVE in broilers is caused by increasing the bioavailability of VE. The in vivo metabolic kinetics of RVE and NVE were investigated. In chickens, as there is lack of relevant reports on pharmacokinetic assays for VE, we established the timing for the pharmacokinetic assay of VE in chickens by referring to the literature of two rat studies (Gong et al., 2012; Parthasarathi et al., 2016). In the study by Gong et al. (2012), rat plasma VE levels returned to baseline at 12 h after feeding nano-emulsion VE, whereas in the research conducted by Parthasarathi et al. (2016), it took 6 hours for the plasma VE levels to return to baseline after feeding normal VE. Our study showed that chickens’ plasma VE levels returned to baseline at 14.5 h. It may be caused by species differences and different animal-rearing conditions. In addition, the results of this study showed that VE prepared in the form of nano-sized emulsion has better oral bioavailability, which was consistent with previous research (Mohd Zaffarin et al., 2020). Simon et al. (2016) indicated that α-tocopherol degradation can be prevented in the stomach environment and intestinal conditions when mixed with PLGA or a combination of PLGA and chitosan (PLGA/chitosan) nanoparticles. This leads to higher plasma bioavailability of α-tocopherol. Nano-formulation have the characteristics of small size and large surface area, which may be responsible for the enhanced VE bioavailability (Desai, 2012).

## Conclusion

In summary, nanotechnology had no effect on the absorption site of VE in the intestine, but significantly improved the antioxidant performance of broilers. This improvement is attributed to the enhanced absorption efficiency and extended half-life of NVE, both contributing to increased bioavailability.

## Abbreviations

ADFI: average daily feed intake
ADG: average daily gain
AUC_0−t_: area under the concentration vs time curve from zero to t
AUC0_−∞_: area under the concentration versus time curve from zero to infinity
BF: the bursa of Fabricius
BW: body weight
C_max_: peak concentration
DEX: dexamethasone
FCR: feed conversion ratio
GSH-Px: glutathione peroxidase
MDA: malonic dialdehyde
MRT: mean residence time
t_1/2z_: half-life of the elimination
NVE: nano vitamin E
RVE: regular vitamin E
SOD: superoxide dismutase
T-AOC: total antioxidant capacity
